# Human papillomavirus in canine serum: evidence from a Chinese study

**DOI:** 10.3389/fvets.2025.1511289

**Published:** 2025-04-30

**Authors:** Yumeng Liu, Lulu Xie, Yimin Zhou, Lin Zhou, Jingshan Bi, Min Zheng, Tian Lan, Wenchao Sun

**Affiliations:** ^1^Institute of Virology, Wenzhou University, Wenzhou, China; ^2^College of Animal Science and Technology, Guangxi University, Nanning, China; ^3^Guangxi Centre for Animal Disease Control and Prevention, Nanning, China

**Keywords:** papillomavirus, human papillomavirus, canine serum, phylogenetic analysis, public health

## Abstract

**Introduction:**

Human papillomaviruses (HPVs) are well-known for causing both benign and malignant epithelial growths in humans, but their occurrence in non-human species is rarely reported. Expanding the understanding of HPV’s host range is essential for assessing its ecological and public health implications.

**Methods:**

We investigated serum samples from dogs collected in Guangxi, China, between 2014 and 2020. PCR screening was performed to detect HPV DNA, followed by sequencing and phylogenetic analysis of the positive amplicons.

**Results:**

HPV DNA was unexpectedly detected in 2 out of 1,226 canine serum samples, yielding a detection rate of 0.16%. Phylogenetic analysis revealed that the sequences clustered with alpha2-HPV78 (GX-70-related) and alpha2-HPV94 (GX-47-related), respectively. Interestingly, both sequences displayed multiple amino acid variations in viral proteins. However, virus isolation was not achieved.

**Discussion:**

The detection of HPV nucleic acids in canine serum suggests a potential presence of HPV in canine hosts. These findings provide new insights into the possible host range of HPV, underscoring the need for further research to assess the virus’s infectivity, transmission dynamics, and implications for both animal and human health.

## 1 Introduction

**Figure 1:**
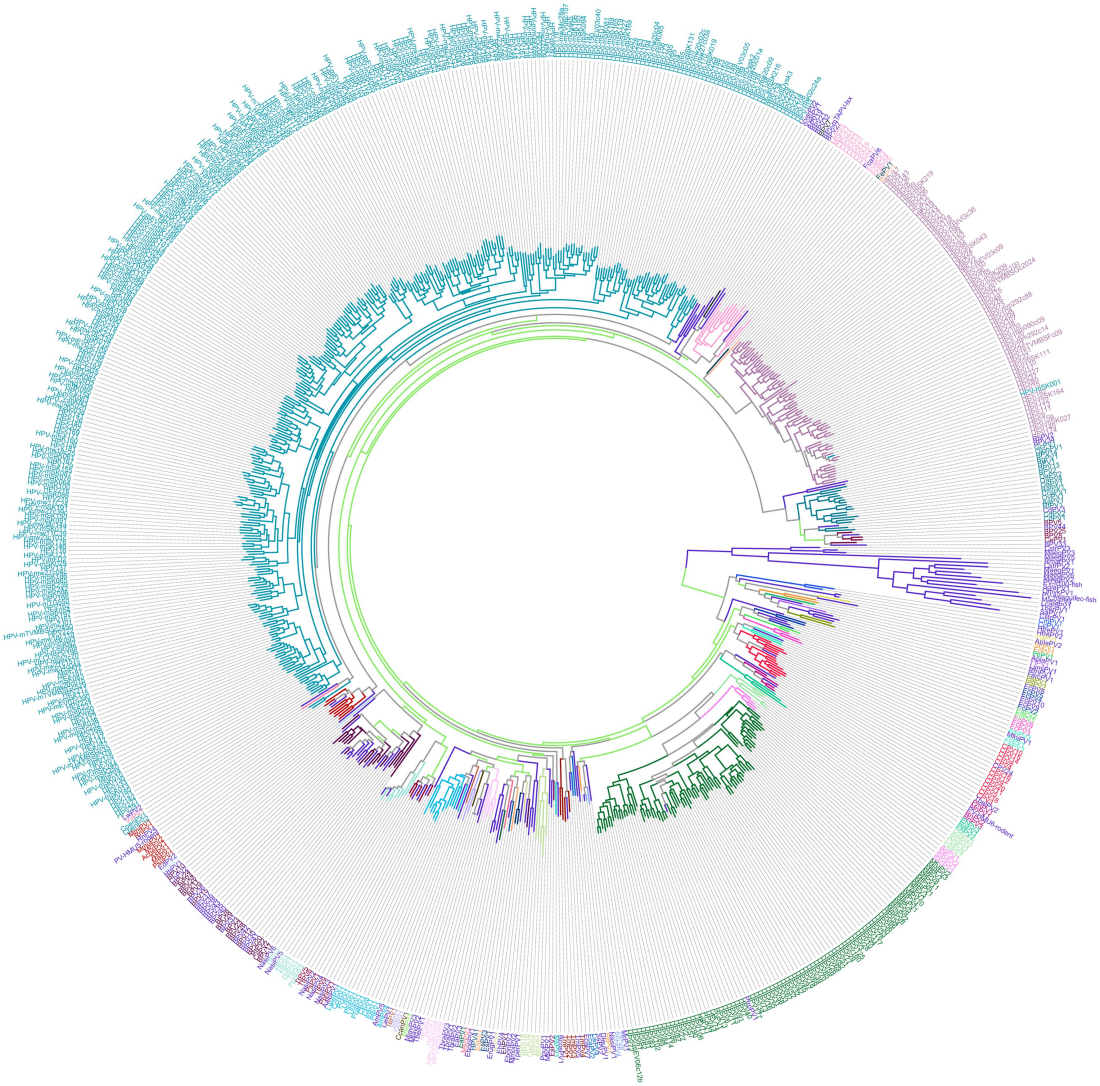
This figure provides a comprehensive view of papillomavirus diversity. Phylogenetic tree of papillomavirus (PV) L1 gene sequences. The L1 nucleotide sequences of all papillomavirus types currently available in the Papillomavirus Episteme (PaVE) database were aligned using MAFFT. A maximum likelihood tree was constructed using PhyML, implementing the GTR + I + G substitution model to account for among-site rate variation and variable substitution rates. This model was selected based on jModeltest analysis. The resulting phylogenetic tree was visualized and displayed using phylotree.js.

Papillomaviruses (PVs) are a large and diverse group of DNA viruses that infect a broad range of vertebrate species, from benign warts to malignant cancers, with human papillomaviruses (HPVs) being particularly well-known for their association with such conditions in humans (1–3). While more than 200 human papillomavirus (HPV) types have been identified and extensively studied for their role in human health (4), papillomaviruses have been detected across a broad range of animal species, reflecting their evolutionary adaptability to diverse hosts (Figure 1) (5). Papillomavirus infections have been documented in species as varied as cattle, horses, cats (6), dogs, avian (7), and even more exotic animals such as giant pandas, pangolins, and whales (8, 9). Despite their generally host-specific nature, the detection of papillomaviruses in a wide variety of animals suggests significant evolutionary diversification within the Papillomaviridae family (4). While HPV has been extensively studied in humans, reports of papillomavirus infections in non-human species are relatively scarce, and few studies have explored the presence of papillomaviruses in other mammals.

While infection models for HPV in dogs and mice have recently been established (10, 11), reports of natural HPV infections in non-human species remain limited. Papillomaviruses have traditionally been regarded as highly host-specific, with minimal evidence of cross-species transmission (12). However, bovine papillomavirus (BPV) has demonstrated its ability to infect a wide range of species beyond cattle (13). BPV infections have been documented in wild animals such as tapirs, giraffes, antelopes, and zebras, as well as domestic animals like horses and donkeys, where it causes equine sarcoids (14). Furthermore, molecular detection of BPV DNA in the placenta and blood of healthy mares and their foals suggests potential vertical transmission, further supporting the notion that PV can cross species barriers (4, 15). Moreover, systematic studies on HPV in domestic animals, particularly dogs, are scarce, despite the close interaction between dogs and humans. Investigating the potential presence of HPV in dogs could provide important insights into the ecological complexity and host range of papillomaviruses.

In this study, we examined canine serum samples collected in Guangxi, China, from 2014 to 2020 and detected HPV DNA in two serum samples. To our knowledge, this is the first report of naturally occurring HPV detected in dogs outside of experimental models, raising important questions about the potential for papillomaviruses to infect a broader range of hosts in natural environments. Although contamination cannot be entirely ruled out, the detection of HPV nucleic acid in canine samples highlights a previously unexplored aspect of HPV ecology. These findings underscore the need for further investigation into the infectivity, transmission dynamics, and broader implications of papillomaviruses for both animal and human health.

## 2 Materials and methods

### 2.1 Papillomavirus DNA detection and PCR

From 2014 to 2020, an extensive surveillance program was conducted in China to monitor rabies antibody prevalence across various species, including domestic pets (dogs and cats), rural dogs, and wild animals such as foxes and raccoons. Serum samples were collected from pets diagnosed in veterinary hospitals and from rural dogs undergoing rabies antibody testing at the Guangxi Animal Disease Prevention and Control Center, China. Serological testing was first conducted to detect antibodies against rabies virus. Subsequently, DNA was extracted from the serum samples, and PCR assays were performed to identify the presence of CPV viral DNA. We unexpectedly detected sequence fragments corresponding to papillomaviruses (PVs) in several serum samples. This unexpected discovery prompted us to perform additional PCR assays specifically targeting conserved regions of the PV genome. The primer sequences used for full-length genome amplification are provided in Supplementary Tables S1, S2. The applied primer sequences and amplification conditions are shown in Table 1. The PCR mixture consisted of 2 μL extracted DNA, 2 μL of the primer pair (10 μM), 25 μL of 2 × Phanta Max Master Mix (Vazyme, Nanjing, China), and 21 μL DNase/RNase-free water. PCR amplification was performed under the following cycling conditions: initial denaturation at 95°C for 3 min; 35 cycles of 95°C for 15 s, 62°C for 15 s, and 72°C for 1 min; followed by a final extension at 72°C for 5 min. After amplification, 15 μL of the PCR products were analyzed by 1.0% agarose gel electrophoresis, and the target bands were purified using a gel extraction kit. Each fragment was amplified and analyzed in triplicate to ensure reproducibility. The PCR products of the three fragments were cloned into the pMD18-T Easy vector (Takara Biotechnology Co., Ltd., Dalian, China) and submitted for Sanger sequencing by Sangon Biotech (Shanghai, China). Among these, those with the highest quality and concentration of viral DNA were chosen to ensure accurate and complete genome assembly. Subsequently, the assembly and editing of viral sequences were carried out using BioEdit software.

**Table 1:** Overview of the different ORFs of the HPVs GX-47 and GX-70, their position in the genome, the length of the proteins they encode, and the other features they contain at the nucleotide (nt) or amino acid (aa) level.

Gene	Positions (nt)		Fragment length		Number of encoded amino acids (aa)		Amino acid differences		Function
	GX-47	GX-70	GX-47	GX-70	GX-47	GX-70	GX-47 (AJ620211)	GX-70 (AB793779)	
E6	57–482	188–508	426	321	142	107	R47H		Associated with the development of epithelial malignancies
E7	485–736	532–783	267	252	89	84		H56P	Re-entry into S-phase cell cycle
E1	749–2,791	796–2,775	2043	1980	681	660	G220H, R501X, I502X, P503X, I581M	D42E, K598T	Viral DNA replication and transcription
E2	2,736–3,854	2,720–3,841	1,119	1,122	373	374	S220R	N247D, W251R, Q291R, N292H	Viral DNA replication, apoptosis, transcriptional repressor of E6/E7
E4	3,340–3,627	3,324–3,614	288	291	96	97	A21E	A51V, K94D	Viral DNA replication
L2	4,380–5,672	4,372–5,721	1,293	1,350	431	450	S13F	A61S, P96A, T112S, N260D, R285K, G296X, G297X, V333I, S349A, A370T, D380N, S381N, V451I	Major capsid protein
L1	5,731–7,242	5,741–7,195	2,862	1,455	954	485	S487W, T502P	I107V, T118S, P180S, N274S	

### 2.2 Sequence assembly and phylogenetic analysis

Open reading frames (ORFs) of the complete genomes were predicted using the ORF Finder tool^1^. Multiple sequence alignments were conducted between the papillomavirus (PV) genomes identified in this study and reference PV sequences from different hosts using the Clustal W method. Percent pairwise identities, based on nucleotide and deduced amino acid sequences, were calculated using SDT3.4. Phylogenetic analysis was performed on the two canine-origin HPV strains as well as 433 reference sequences, which were obtained from the Papillomavirus Episteme (PaVE) database^2^. The E6 and L1 gene sequences from the GX-70 (HPV-78) and GX-47 (HPV-94) canine HPVs were aligned with reference genomes using ClustalW. Subsequently, maximum-likelihood phylogenetic trees were constructed with MEGA7, using midpoint rooting, and visualized with iTOL^3^. The best-fit model of nucleotide substitution, determined based on the Akaike Information Criterion (AIC), was GTR + G + I. Both maximum-likelihood and neighbor-joining trees were generated with a bootstrap value of 1,000 (25). All phylogenetic analyses were conducted using MEGA 7.0.

## 3 Results

### 3.1 PV discovery and genome annotation

**Figure 2:**
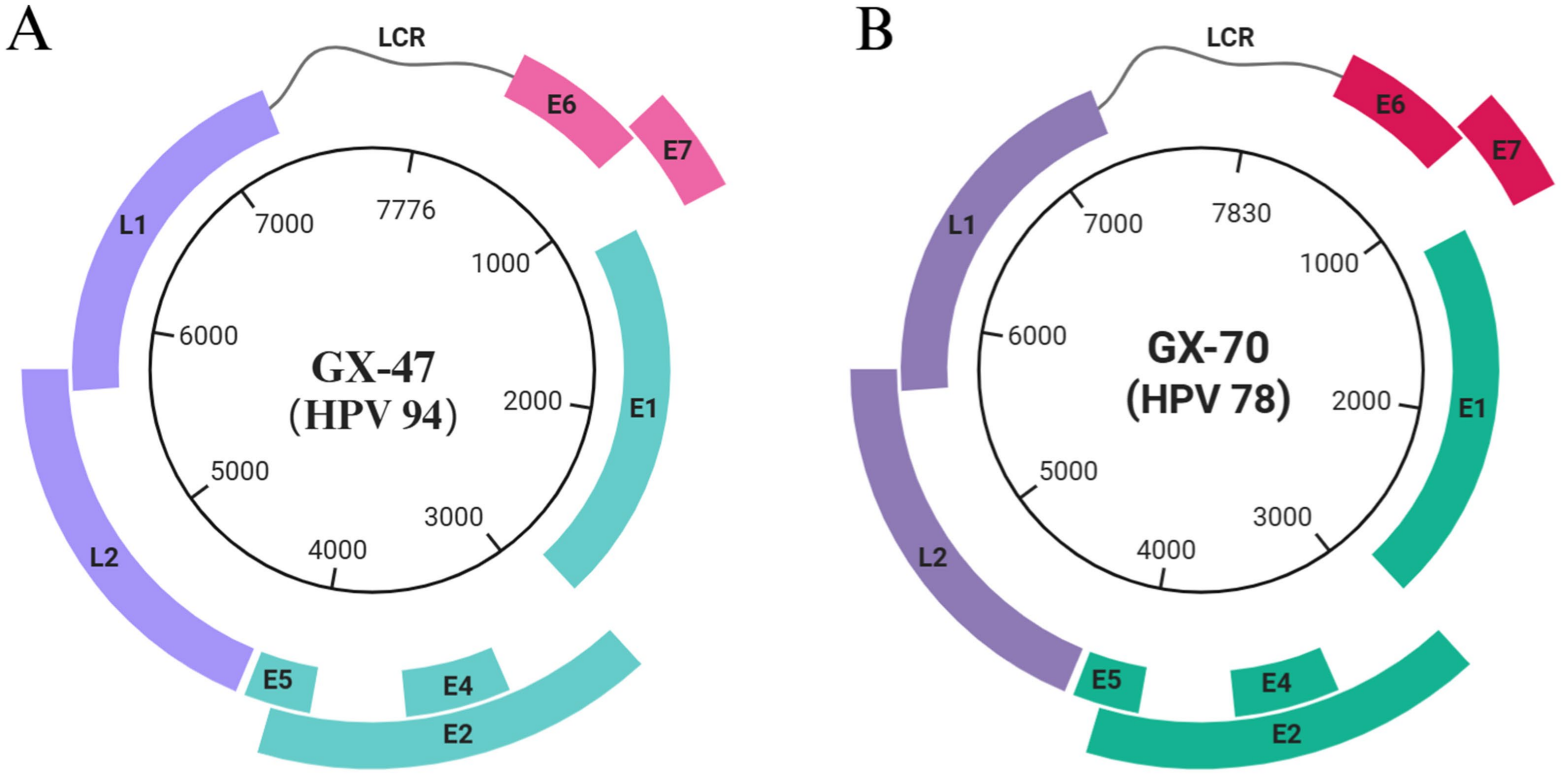
Genome organization of the two papillomaviruses identified in this study. **(A)** Circular genome organization of GX-47 (HPV-94); **(B)** Circular genome organization of GX-70 (HPV-78). The scale inside the circles represents nucleotide positions of the viral genome.

We detected two HPV-positive samples from 1,226 canine serum samples, yielding a detection rate of 0.16% (2/1226). Whole-genome sequencing of these samples revealed two distinct viral genomes, GX-70 (7,776 bp) and GX-47 (7,830 bp). Using the NCBI ORF prediction tool, we identified key papillomavirus open reading frames (ORFs), including early genes (E6, E7, E1, E2, E4) and late genes (L1, L2). The genome structures of GX-70 and GX-47 are illustrated in Figure 2, depicting the positions and sizes of each ORF, which provides a comprehensive comparison of the genomic organization of the two viral strains.

Detailed analysis of the open reading frames (ORFs) revealed multiple key amino acid substitutions between the two strains, including R47H in the E6 gene of GX-47 and H56P in the E7 gene of GX-70. These substitutions may have profound effects on viral replication, transcriptional regulation, and host-pathogen interactions, given the critical roles of E6 and E7 in the viral life cycle. Table 1 summarizes these mutations alongside the nucleotide positions, fragment lengths, and number of encoded amino acids for each ORF. The data indicate potential functional divergence between GX-47 and GX-70, laying a foundation for future studies to explore the implications of these mutations on viral behavior and pathogenicity.

### 3.2 Phylogenetic analysis

**Figure 3:**
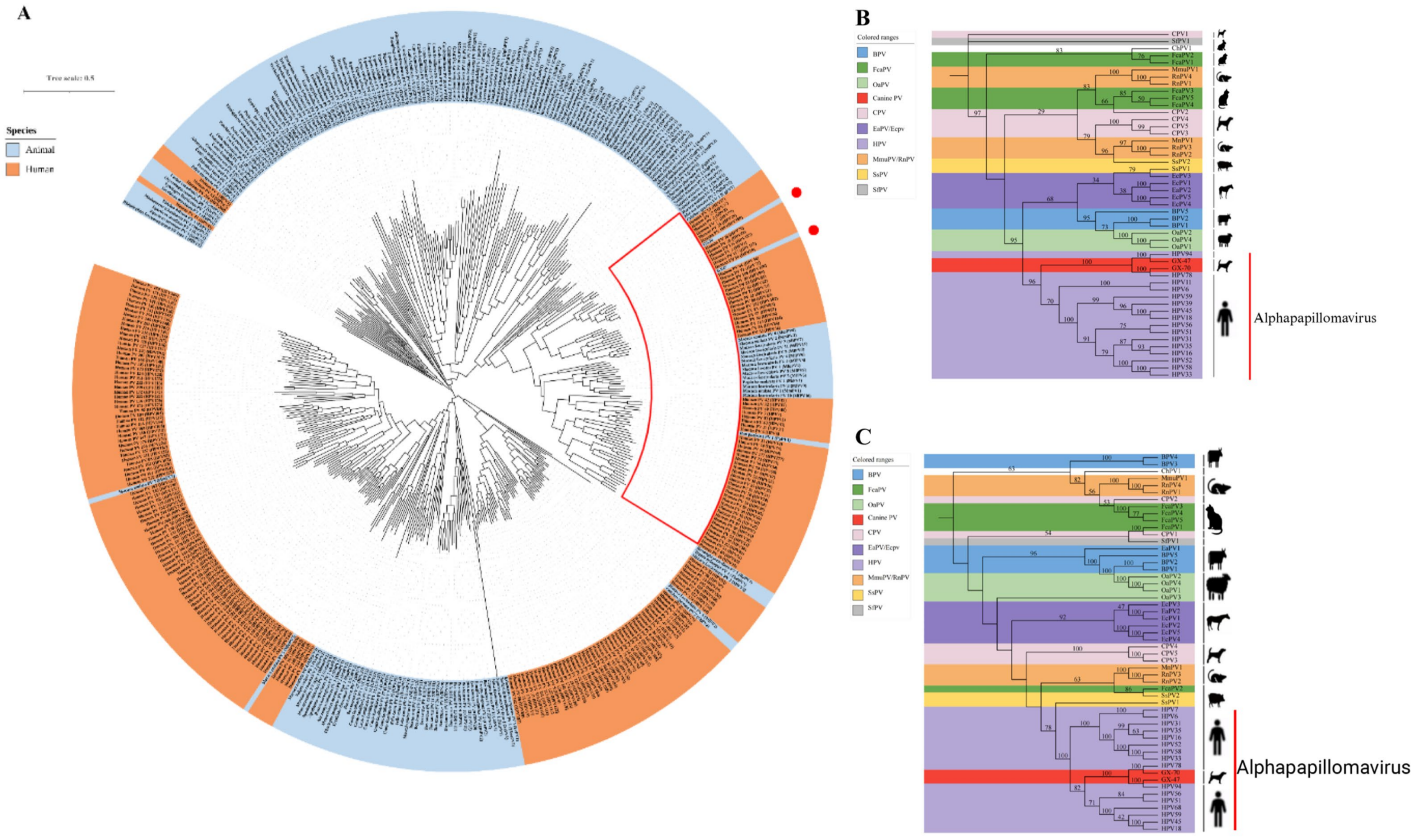
**(A)** The maximum likelihood (ML) tree of papillomavirus based on whole-genome sequences (downloaded from PAVE database), with a tree visualized with iTOL (https://itol.embl.de) and midpoint rooted. Two Chinese canine-origin HPV GX-47 and GX-70 strains (marked with a red circle) in the study. The red box highlights the clade belonging to the Alphapapillomavirus genus. **(B)** Phylogenetic trees generated with L1 regions of the papillomaviruses in canine serum. **(C)** Phylogenetic trees generated with E6 regions of the papillomaviruses in canine serum. The red lines in **(B)** and **(C)** indicate the Alphapapillomavirus genus. (BPV – *Bos taurus* papillomavirus; CPV – canine papillomavirus; EaPV – *Equus asinus* papillomavirus; EcPV – *Equus caballus* papillomavirus; FcaPV – Felis domesticus papillomavirus; HPV – human papillomavirus; MnPV – *Mastomys natalensis* papillomavirus; OaPV – *Ovis aries* papillomavirus; SsPV – *Sus scrofa* papillomavirus; SfPV – *Sylvilagus floridanus* papillomavirus).

To understand the evolutionary relationships of GX-70 and GX-47, we conducted a maximum-likelihood phylogenetic analysis based on their whole-genome sequences. The analysis revealed that GX-70 clusters closely with HPV-78, while GX-47 shows slight divergence from HPV-94. Both strains are positioned within the Alphapapillomavirus 2 clade, aligning with their closest human relatives (Figure 3A). For further validation, we also analyzed the L1 and E6 genes. The L1 gene encodes the viral capsid protein, and the phylogenetic tree for L1 mirrored the overall whole-genome phylogeny (Figure 3B). Similarly, the E6 gene, which is associated with epithelial malignancies, showed consistent evolutionary patterns with the full genome and L1-based trees (Figure 3C). This consistency across different gene segments supports the close evolutionary relationship between GX-70, GX-47, and their related strains, reinforcing their classification within the Alphapapillomavirus lineage.

### 3.3 Canine-origin HPV sequence identity and amino acid mutations

**Figure 4:**
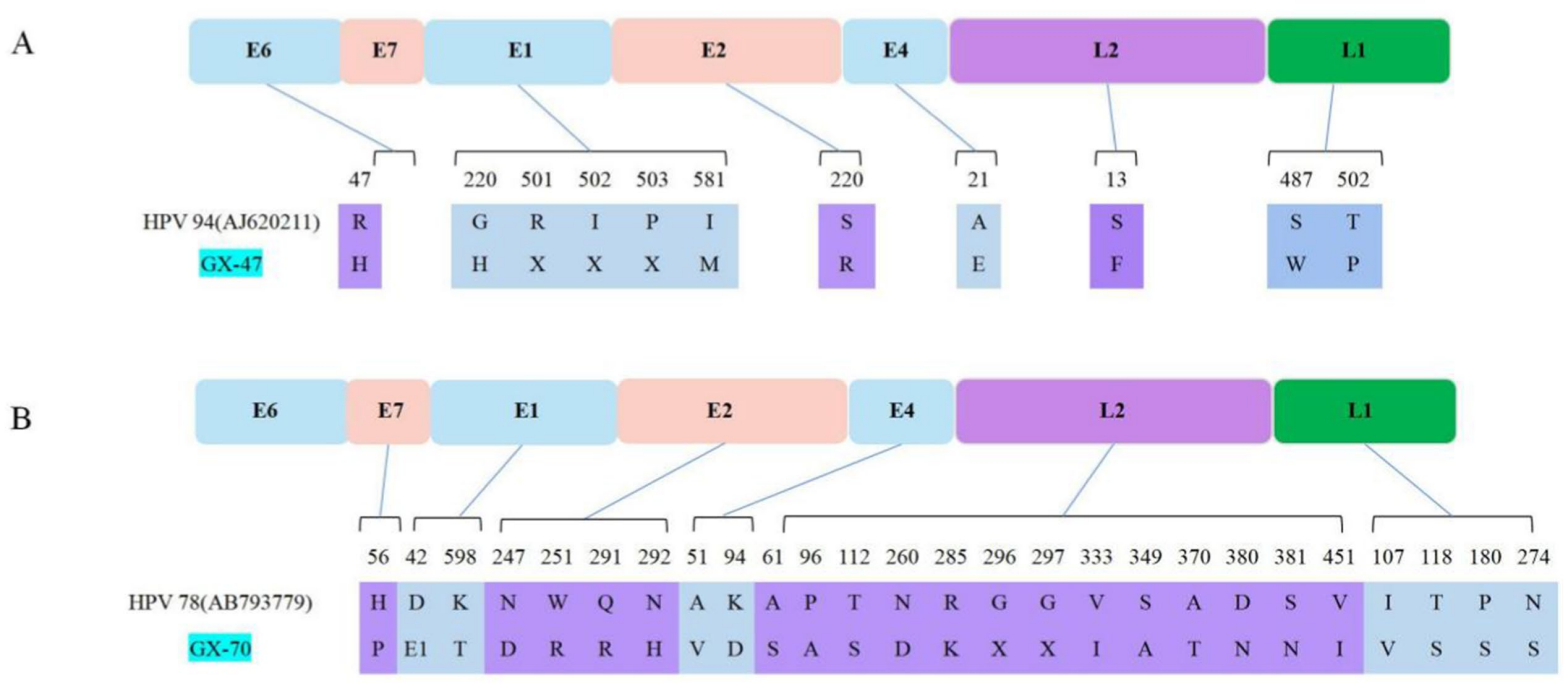
Schematic representation of amino acid substitutions identified in the GX-47 **(A)** and GX-70 **(B)** strains of papillomavirus compared with their respective reference genomes (HPV 94 [AJ620211] and HPV 78 [AB793779], respectively). Colored boxes represent viral open reading frames (ORFs), with corresponding positions and amino acid changes shown below each gene. Mutations are mapped to their respective positions in the viral proteins, highlighting potential differences in functional domains.

The comparative genomic analysis revealed that the genomes of the two HPV-positive canine samples shared a nucleotide identity of 82.1%. The nucleotide identity of GX-47 with the reference strain AJ620211 was as high as 99.4%. In contrast, the nucleotide identity between the GX-70 strain and the reference gene HPV-78 (No. AB793779) was 98.4% (Figure 4). However, the amino acid sequence homology between these two strains and canine papillomavirus (CPV) ranged from 44.7 to 49%.

The closest related sequence in GenBank, HPV-94 (No. AJ620211), exhibited multiple amino acid differences when compared to GX-47 and GX-70. As illustrated in Figure 4A, the GX-47 strain contains a key substitution in the E6 gene (R47H) as well as notable changes in the E2 (S220R) and L2 (S13F) proteins, which may impact viral replication and host-pathogen interactions. These substitutions may indicate a potential adaptation mechanism within GX-47. Similarly, in Figure 4B, the GX-70 strain is shown to have 13 novel substitutions in the L2 protein at positions 61, 96, 112, 260, 285, 296, 297, 333, 349, 370, 380, 381, and 451, as well as mutations in E7 (H56P) and E1 (D42E, K598T). These mutations, especially in the L2 protein, could affect capsid stability, immune evasion, and viral DNA packaging. Substitutions in the N-terminal region of L2 (positions 1–200) may impact capsid assembly and immune recognition, while mutations in the central and C-terminal regions (positions 200–450) could alter receptor interactions and viral entry mechanisms. These observed differences, presented in Figure 4, suggest that GX-70 may have undergone evolutionary adaptations specific to the canine host.

## 4 Discussion

This study presents the first molecular evidence of human papillomavirus (HPV) strains in canine serum, revealing an unexpected dimension of papillomavirus (PV) biology. Serum has proven to be a valuable sample for detecting viral nucleic acids and antibodies in various canine virus studies. Previous research has identified several novel viruses in canine serum, such as canine bufaviruses (CBuVs) (16), porcine circovirus 3 (PCV3) (17), Torque teno canis virus (TTCaV) (18), and canine circovirus (CanineCV) (19, 20). The detection of papillomaviruses in serum, however, is particularly noteworthy, as PVs are traditionally associated with epithelial tissues, primarily being detected in skin or mucosal lesions. The presence of HPV nucleic acids in dogs suggests either a viremic phase or a broader tissue tropism, challenging the current understanding of PV biology (21).

While infection models for HPV in dogs have been established, these experimental models have primarily focused on viral pathogenesis under controlled conditions. Our detection of HPV in naturally collected canine serum highlights a previously unexplored aspect of HPV ecology, suggesting the potential for systemic infection beyond these experimental settings. This finding raises important questions about whether HPV can establish natural infections in dogs, a species that shares close proximity with humans.

Environmental contamination from human-derived HPV cannot be entirely excluded. However, the detection of papillomavirus DNA in blood across other species, including horses and cattle, supports the hypothesis of a viremic phase in certain PV infections (22). This finding prompts further investigation into whether HPV may circulate beyond epithelial tissues in non-human hosts, potentially expanding the virus’s transmission routes.

Our findings also mirror the well-documented cases of cross-species transmission involving bovine papillomavirus (BPV), which infects animals such as horses, zebras, and donkeys, and is implicated in the development of equine sarcoids (23). BPV’s ability to cross species boundaries suggests that HPV may similarly possess this potential under certain conditions, though such cross-species transmission of HPV remains speculative. The detection of viral strains in dogs—animals in close, regular contact with humans—raises significant questions about zoonotic risk and potential viral adaptation to new hosts (24).

A significant limitation of this study is the inability to isolate live virus or confirm replication in canine cells. Although HPV DNA was detected in serum samples, without evidence of viral replication or protein expression in host cells, we cannot conclusively determine whether these viruses established productive infections. We attempted virus isolation using conventional cell culture techniques with multiple cell lines, but no cytopathic effects or viral replication were observed. This may be due to the lack of appropriate permissive cell models or the possibility that HPV does not actively replicate in canine blood cells. To address this limitation, future studies should explore the use of primary canine epithelial cells, organoid systems, or *in vivo* models to assess viral infectivity, tissue tropism, and replication competence. These approaches will help clarify whether the presence of HPV DNA in canine serum represents a transient exposure, systemic spread, or a true active infection.

Despite these limitations, the detection of HPV DNA in canine serum raises important questions regarding viral transmission dynamics and host range. Given the increasing evidence of PVs in various animal species and the close interaction between dogs and humans, further research is essential to explore the possibility of interspecies transmission. Established HPV infection models in dogs provide a valuable platform for studying viral replication, immune responses, and the broader health implications in canine hosts.

While evaluating host immune responses is critical for understanding virus–host interactions, our study did not include serological analysis. This was primarily due to the lack of validated serological assays for detecting antibodies against HPV L1 or E6 proteins in dogs. Currently, no standardized protocols or reliable canine-specific secondary antibodies are available to facilitate such measurements. We acknowledge this limitation and propose that future studies prioritize the development of species-specific serological tools to enable comprehensive assessment of humoral immune responses in exposed animals.

In conclusion, this study provides the first molecular evidence of HPV in canine serum, suggesting that papillomaviruses may have a broader host range than previously recognized. Further research is critical to clarify the mechanisms of HPV transmission in dogs and assess the potential health risks for both canines and humans.

## Data Availability

The datasets generated and analyzed during this study are publicly available in the GenBank repository. The complete genome sequences of the two canine-origin human papillomavirus (HPV) strains have been deposited under the following accession numbers: GX-47 (PV384155) and GX-70 (PV384154). Additional data supporting the findings of this study are provided in the Supplementary Material.

## Footnotes

^1^ https://www.ncbi.nlm.nih.gov/orffinder/

^2^ https://pave.niaid.nih.gov/

^3^ https://itol.embl.de
