# Supplementary material for: Human papillomavirus in canine serum: evidence from a Chinese study
Source: Front Vet Sci. 2025 Apr 30;12:1511289. doi: 10.3389/fvets.2025.1511289 (PMC12075875; doi:10.3389/fvets.2025.1511289)
Supplement: Supplementary file 1 [file Table_1.docx]

**Human Papillomavirus in Canine Serum: Evidence from a Chinese Study**

**Supplementary materials**

Table S1. List of primers used for PCR amplification of GX-70 sequences in this study.

| Primer name | Sequence (5'–3') |
| --- | --- |
| 70-1F | 5' TTATAAACTATAATCAACAGAATAA 3' |
| 70-13F | 5' ATCAACAGAATAAGAAAGTAGGGAGGGAC 3' |
| 70-612R | 5' ATCTAATTGCTCATTGCATAGTAGAGGGA 3' |
| XS2-532F | 5' GGTCCGCATCCCACAATAAAAGAAATAGA 3' |
| XS2-1560R | 5' ATACACCCCAAACACACACACAACCCAAT 3' |
| 70-1518F | 5' AACTGCATGTTGTGATTGGGTTGTGTGTG 3' |
| 70-2720R | 5' TTTTCCTCCTCCTCTTCAGGATCGGTAAG 3' |
| 70-2518 | 5' ATAACAACCAACATAAATCCGTTAGAGGA 3' |
| 70-3992 | 5' CCCAGGTACAGACACAGCACAATAGCCAG 3' |
| 70-3912F | 5' GTTGCTGCTTTGTTTGTTTTGGCTGTGTG 3' |
| 70-5348R | 5' AGTTCGATGTCCTCTGTGATAGCTATGGG 3' |
| 70-5304F | 5' TTATCATGACCTCAGTCCCATAGCTATCA 3' |
| 70-6622R | 5' ACTATACACCGCACTACCAATAACATCCC 3' |
| 70-6432F | 5' ATTTGTCAGTCTGTTTGTAAATATCCTGA 3' |
| 70-7824R | 5' GTAGAAAGAGAGGTGTAGCAACTGCATTA 3' |
| 70-550F | 5' AAAGAAATAGAATTGAGTCTTGCACCAGA 3' |
| 70-1680R | 5' TGCCCGTTTATACCTTAAAAGCATTAGTA 3' |
| 70-582F | 5' CGTCCCTCTACTATGCAATGAGCAATTAG 3' |
| 70-1600R | 5' TTGGCTGTATTAGTGTTTTTATGCCCTCT 3' |
| 70-1526F | 5' GTTGTGATTGGGTTGTGTGTGTGTTTGGG 3' |
| 70-2638R | 5' ATGTGTACAGTGGCTCTCCTTGCGTTGTT 3' |
| 70-1460F | 5' ATGTGTACAGTGGCTCTCCTTGCGTTGTT 3' |
| 70-2564R | 5' AAATACTTCCATCTCTCATCCTCTAACGG 3' |
| 70-2522F | 5' CAACCAACATAAATCCGTTAGAGGATGAG 3' |
| 70-3768R | 5' CATGTATACCATATTGTCACAAACGCTGC 3' |
| 70-2518F | 5' ATAACAACCAACATAAATCCGTTAGAGGA 3' |
| 70-3734R | 5' TTTCAGATTCACAGGACCACCGCCAAGTG 3' |
| 70-3616F | 5' AATACACTTACGAGGTGATCCTAATAGTT 3' |
| 70-4786R | 5' CATTGTCATTACTGGGTGTTATGTCTAAC 3' |
| 70-3572F | 5' GCATACACAGTCAGGCAACGACATTACCC 3' |
| 70-4730R | 5' GTCACCTCAAAACCACCCGTGCCAGAGAA 3' |
