# Supplementary material for: Human papillomavirus in canine serum: evidence from a Chinese study
Source: Front Vet Sci. 2025 Apr 30;12:1511289. doi: 10.3389/fvets.2025.1511289 (PMC12075875; doi:10.3389/fvets.2025.1511289)
Supplement: Supplementary file 2 [file Table_2.docx]

**Human Papillomavirus in Canine Serum: Evidence from a Chinese Study**

**Supplementary materials**

Table S2. List of primers used for PCR amplification of GX-47 sequences in this study.

| Primer name | Sequence (5'–3') |
| --- | --- |
| 47-1F | 5' CGGTGCGACCGAATGGGGTACATAT 3' |
| 47-21F | 5' CATATAAAACAAGGCACTTACAGACTGGC 3' |
| 47-638R | 5' TACACTTTGGACATACTGTTACCACCCTA 3' |
| 47-602F | 5' CAAGCGTATAGGGTGGTAACAGTATGTCC 3' |
| 47-1847R | 5' TTTGCCTAACTATCCACTCTGGTGTCTCT 3' |
| 47-1694F | 5' GCCAAGAACAGAGAAACAGTAGCAAAAGG 3' |
| 47-3020R | 5' TCTCAATGTCCAGGATTCATGTGCATAGG 3' |
| 47-2804F | 5' CAACAAACTTGAGGACCAGATCACGCATT 3' |
| 47-3958R | 5' CAAAACAGACACAGCAGCAGCACAAAAAA 3' |
| 47-3802F | 5' TTCTCAATGTTGTAAAAGTACCCCCTGGC 3' |
| 47-5096R | 5' ATCAAATGTTAAAAAAGTGGACGGACGTG 3' |
| 47-5058F | 5' GCATTTCTGTCACGTCCGTCCACTTTTTT 3' |
| 47-6284R | 5' CAGTCGCCCGGTGCAGGCGCCGACCTATT 3' |
| 47-6240F | 5' CAAGGGGACCCCGTGCAATAGGTCGGCGC 3' |
| 47-7736R | 5' GCACATTTTATTGGCAGGGCAACCTAGAA 3' |
| 47-7776R | 5' GCAGCGACCGGATTAGGTGTGTCTT 3' |
| 47-2912F | 5' GCCACCTCTTAGTGTAACTAAAGCCAAGG 3' |
| 47-3938R | 5' CACAAAAAAAGCCCAGCAATGTAGTCTAC 3' |
| 47-2950F | 5' CCATTGAAGTGCATGTAGCTTTACAGCAA 3' |
| 47-4138R | 5' GGGCTCAAACAACAATACAGTATGGTCAC 3' |
| 47-2912F | 5' GCCACCTCTTAGTGTAACTAAAGCCAAGG 3' |
| 47-3938R | 5' CACAAAAAAAGCCCAGCAATGTAGTCTAC 3' |
| 47-2864F | 5' GTACAAAGCAAGAGAATGTGGACTGACAC 3' |
| 47-3930R | 5' AAGCCCAGCAATGTAGTCTACACAAATAA 3' |
|  |  |
